# Creating a Bot-tleneck for malicious AI: Psychological methods for bot detection

**DOI:** 10.3758/s13428-024-02357-9

**Published:** 2024-04-01

**Authors:** Christopher Rodriguez, Daniel M. Oppenheimer

**Affiliations:** Department of Social and Decision Sciences, Carnegie Mellon University, 5000 Forbes Avenue, BP 208, Pittsburgh, PA 15213 USA

**Keywords:** Bot detection, Cybersecurity, Data validation, Human-computer interaction, Survey design

## Abstract

The standard approach for detecting and preventing bots from doing harm online involves CAPTCHAs. However, recent AI research, including our own in this manuscript, suggests that bots can complete many common CAPTCHAs with ease. The most effective methodology for identifying potential bots involves completing image-processing, causal-reasoning based, free-response questions that are hand coded by human analysts. However, this approach is labor intensive, slow, and inefficient. Moreover, with the advent of Generative AI such as GPT and Bard, it may soon be obsolete. Here, we develop and test various automated, bot-screening questions, grounded in psychological research, to serve as a proactive screen against bots. Utilizing hand coded free-response questions in the naturalistic domain of MTurkers recruited for a Qualtrics survey, we identify 18.9% of our sample to be potential bots, whereas Google’s reCAPTCHA V3 identified only 1.7% to be potential bots. We then look at the performance of these potential bots on our novel bot-screeners, each of which has different strengths and weaknesses but all of which outperform CAPTCHAs.

## Introduction

A “Completely Automated Public Turing test to tell Computers and Humans Apart” (CAPTCHA), is a small challenge task designed to differentiate humans from machines, such as deciphering distorted letters and numbers or identifying common objects in natural scenes. If AI systems cannot overcome a particular CAPTCHA, then it is a viable method of differentiating bots from humans. However, from their very inception, computer scientists viewed CAPTCHAs as having value regardless of whether they remain unsolved by AI. Once AI systems do overcome the task, the CAPTCHA has served the purpose of helping in the development of smarter algorithms that could solve what was a previously unsolved AI problem (cf. von Ahn et al., 2003). However, to a social scientist trying to ensure high data quality from online experiments, a cybersecurity expert trying to curb phishing or bots’ spread of misinformation, or Taylor Swift’s ticketing agent trying to identify bots to weed out bulk buying and reselling, when an AI system solves a CAPTCHA it is not a “win.” Rather, it makes it harder to distinguish between AI and humans.

Detecting bots has important ramifications. Crowdsourcing platforms like Amazon’s Mechanical Turk (MTurk) have been an undeniably powerful tool that social science researchers can use to gather large, representative, and relatively cheap samples. While these platforms work wonders for efficiency with regards to time, money, and logistics, lab studies have one very distinct advantage: you know that your participant is human.

In the summer of 2018, researchers began to find empirical evidence that there was a notable decrease in data quality on MTurk (Chmielewski & Kucker, 2019), with an increase in free response answers that were irrelevant to the question and riddled with errors (Moss & Litman, 2018). As standard methods for detecting bots began to fail, data from crowdsourced platforms became increasingly unreliable, culminating in a recent article documenting a study with less than 3% of the ‘participants’ providing valid answers (Webb & Tangney, 2022). The inability to detect bots has rendered a powerful tool in the social scientists’ toolbox ineffectual.

Social scientists running surveys on crowdsourcing platforms are not the only group that fight bots daily. Cybersecurity experts wage war against bots to help curb the spread of misinformation online (for a review, see Ferrara, et al., 2016). For example, one study recently found that approximately 20% of the content generated on Twitter germane to the 2016 presidential election was generated by bots, and that such content notably detracted from the quality and accuracy of political discourse (Bessi & Ferrara, 2016).

While several countermeasures have been proposed for identifying bots, advances in AI have undermined the effectiveness of these tools. Researchers have documented that two of the primary status quo bot detection techniques, CAPTCHAs and honeypots, are less effective at detecting bots than they once were (Godinho et al., 2020; Teitcher et al., 2015).

For example, the earliest CAPTCHAs revolved around deciphering distorted text. However, these CAPTCHAs spurred the development of new computer-vision techniques that led to the cracking of these elementary text distortion tasks (e.g., Mori & Malik, 2003; Huang et al., 2008). CAPTCHAs evolved in difficulty with new types of distortions, including 3D and animated text versions. In 2014, Google developed a neural network that was able to break one of the hardest text-based CAPTCHAs at the time (reCAPTCHA V1) 99.8% of the time (Goodfellow et al., 2013).

An alternative to text-based CAPTCHAs are image-based CAPTCHAs which involve individuals completing an image classification or recognition task. Image-based CAPTCHAs come in various shapes and sizes. These include, but are not limited to, (1) click-based where individuals must click the part of the non-segmented image indicated by the prompt (e.g., click the shoes in the image below), (2) sliding image-based where individuals must use a slider to solve the challenge (e.g., rotating the image so that it is upright), (3) drag and drop in which participants must combine or reorder image pieces to form a coherent image (e.g., inserting a puzzle piece into the correct spot to complete an image), (4) selection-based where individuals are presented with many small panels of images and asked to select the images that fit a particular classification (e.g., select all of the traffic lights; or select all of the images of dogs) (Guerar et al., 2021). Just as with text-based CAPTCHAs, researchers have now developed programs that solve image-based CAPTCHAs. Notably, Sivakorn et al. (2016) developed a method of breaking Google’s reCAPTCHA V2 and Facebook’s image-based CAPTCHA 70.78 and 83.5% of the time, respectively. Zhao et al. (2018) were able to break reCAPTCHA V2 79% of the time.

Another prominent approach to detecting bots involves analyzing browsing history and cookies of the user (e.g., Google’s reCAPTCHA V2 & V3) to see if the browsing environment appears “human.” Based upon this observed browsing environment, Google’s reCAPTCHA V3 creates a score ranging from 0.0 to 1.0, where 0.0 is very likely a bot and 1.0 is very likely a human. Users of this CAPTCHA can then subsequently decide what score threshold they will use to identify likely bots (as a default, a threshold of 0.5 is commonly used). Unfortunately, bots are easily capable of spoofing the browser history and cookies of an actual human (Sivakorn et al., 2016), and the solution to this problem is not immediately obvious in many circumstances. For instance, one might initially think to combine reCAPTCHA V3 with a common visual CAPTCHA, shown only to participants with low reCAPTCHA V3 scores. However, this creates two potential ways for bots to get through. A bot that is sophisticated enough to mimic browsing history will not be presented with a CAPTCHA that it may or may not have failed. On the other hand, a bot that fails to mimic browsing history may very well be able to complete a more standard CAPTCHA. Thus, this gives bots with low scores a second chance to get through.

Requiring answers to involved free-response questions is a stronger way to identify bots (Storozuk et al., 2020). However, manually confirming that each individual participant is human can be a tedious, slow, and expensive process, and errors can be made, especially for large samples. Moreover, it does not allow for bots to be screened upon entry into the system. Thus, for research purposes, scholars using crowdsourcing platforms need massively increased sample sizes to maintain power after eliminating contaminated data and for cybersecurity purposes, the information has already been spread before bots are detected.

Common methods of bot detection in cybersecurity rely on machine learning to try to identify content most likely to have been generated by bots, and either delete or attach warnings to such content (see Davis et al., 2016 for a representative example). Similar to identifying bots via free response questions, this means the bots are not detected until after information is disseminated, and by that point, such information could be propagated by other bots or humans alike.

The purpose of this paper is to develop a variety of questions, grounded in the literature on human vs. machine intelligence, which will be challenging for bots and easy for humans. While we recognize that it is likely inevitable that these techniques will fall prey to advances in machine learning as previous generations of CAPTCHAs have, we hope to provide tools that can have current practical application for bot detection and serve as inspiration for future generations of bot-detection methodologies. Moreover, we hope the introduction of these tools will help AI researchers improve algorithms (cf. von Ahn, et al., 2003), and will help better elucidate differences between machine and human intelligence.

## Methods

### Recruitment

Participants were recruited from MTurk utilizing CloudResearch (Litman et al., 2017). Notably, while CloudResearch and MTurk provide a variety of security measures that can be employed in order to increase data quality, given our purpose of comparing human and bot performance on novel attention checks, we did not employ these settings in order to ensure sizable groups of both humans and bots to make such comparisons. Specifically, we elected not to (1) solely include CloudResearch Approved Participants, (2) block low-quality participants, (3) verify worker country location, or (4) block suspicious geocodes. However, we did elect to block duplicate IP addresses to ensure we did not simply have the same bot taking the survey repeatedly. We aimed to recruit 900 participants based in the United States in batches of 100 participants per day. Participants were compensated $2.25 for their completion of the survey. Our total sample consisted of 906 individuals. Our study was approved by the Carnegie Mellon University Institutional Review Board (Protocol ID: STUDY2021_00000234).

### Study outline

Upon entering the study and completing the informed consent form, participants were presented with our bot detection questions (see section titled “Bot-Detection Questions” below for specifics regarding bot-detection questions). Participants completed 22 of these questions. These questions were presented in random order, and the specific questions a given participant was shown was a random subset from a larger question pool. After completing these questions, they were presented with our four identification questions (see section titled “Identification questions” below for specifics regarding identification questions). These four questions were shown in random order to participants. After the identification questions, participants were presented with a demographic questionnaire. Finally, they were presented with Google’s reCAPTCHA V3 after which they were debriefed.

### Identification questions

**Fig. 1:**
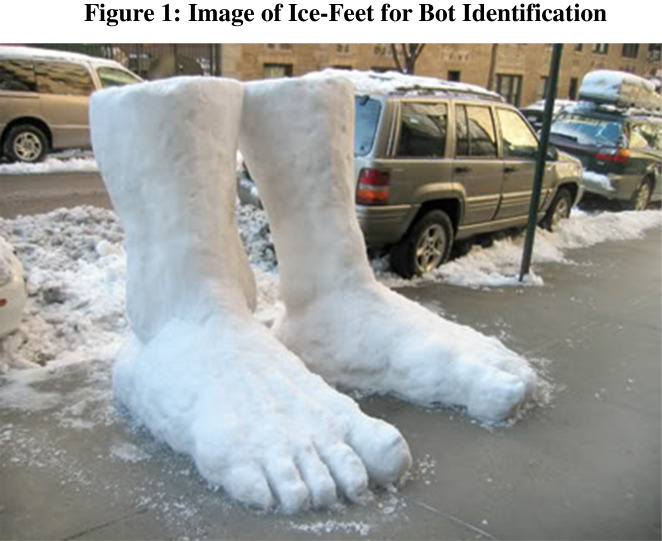
Image of a sculpture by G. Augustine Lynas (2010) that was presented for one of our identification questions. Specifically, participants were asked, “Using a sentence or two, please describe what is likely to happen to the foot below on a warm, sunny day.” While the answer, “they will melt,” is trivial to arrive at for us humans, there are many steps required to arrive at this answer. In order to successfully answer the question, one needs to (i) understand that the feet in the picture are different from normal feet because they are made of ice, (ii) understand that the feet will melt on a warm, sunny day, and (iii) articulate this fact in a coherent-free response

The purpose of these questions is to accurately identify participants as either bot-like (low quality) or human-like (high quality) to serve as a comparison standard for both the CAPTCHA and our automated attention checks. Identification questions involve processing, understanding, and responding, in free response format, to a question about an “odd” picture. For example, one of the questions presented participants with a picture showing a large ice sculpture of two feet (Fig. 1), with the prompt, “Using a sentence or two, please describe what is likely to happen to the foot below on a warm, sunny day.” Of course, the answer is that the feet will melt. However, in order to successfully answer the question, one needs to (i) understand that the feet in the picture are different from normal feet because they are made of ice, (ii) understand that the ice will melt on a warm, sunny day, and (iii) articulate this fact in a coherent free response. These free responses were then examined by humans for relevance and coherence which can provide insight to the identity of the respondent.

In total, we designed four questions to serve as the identification questions. Three human RAs were trained to read and code answers to identification questions and score each response. Responses were given a score of 0 if the response was bot-like, a score of 1 if the response was human-like, and a score of 0.5 if the response could not be clearly categorized. For instance, with regards to the foot ice-sculptures, responses were graded as human-like (1) if they reference anything to do with melting, dissolving, creating puddles, collapsing, or similar articulations of the understanding that the warm sun will melt the ice sculpture. In many cases, bot-like (0) responses are clearly identifiable because they show a fundamental misunderstanding or disregard for the question at hand (e.g., “SNOW” or “At a sunny day at the beach, the top of the sand is warm. The radiation from the sun heats up the surface of the sand, but sand has a low thermal conductivity, so this energy stays at the surface of the sand.”). Responses received a 0.5 if they approached human-like responses, but were slightly off, in terms of grammar and/or terminology (e.g., “They will start leak. Leaky feet!”). In many cases, 0.5 s could likely have been written by non-native English speakers.

**Fig. 2:**
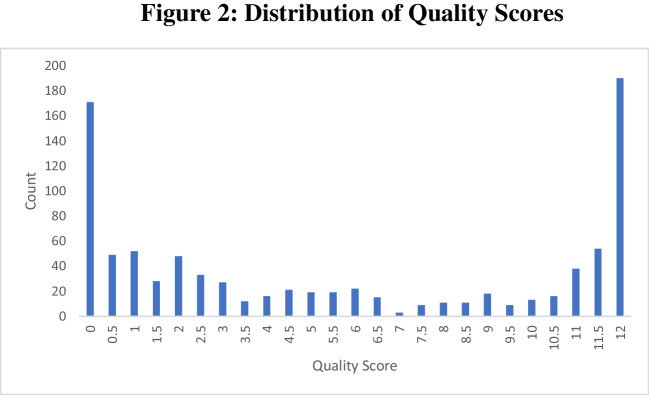
Full distribution of quality scores in our sample. For each free-response identification question, three coders rated a participant’s response as bot-like (0), human (1), or unsure (0.5). A participant’s quality score is derived by summing the ratings of all coders for all four responses that the participant gave to the identification question. An LQP has a score of 0 and exhibits bot-like tendencies, and an HQP has a score of 12 and exhibits human-like tendencies. Participants tended to have a quality score at one of the two extremes of this scale

Following a set of rules for each of the four identification questions, the three RAs evaluated every response given by participants. In total, this means that each participant received 12 evaluations (three raters scoring the participants’ responses to each of four images; 10,872 evaluations in the entire dataset). These evaluations were found to be highly internally consistent with a Cronbach’s alpha = 0.959. Subsequently, we summed each of the 12 evaluations for each participant to make a composite “quality score” for each individual. In our system, a quality score of 0 corresponds to 12 evaluations of 0. We call participants with a quality score of 0 “low-quality participants” (LQPs). Similarly, a quality score of 12 corresponds to 12 evaluations that the participant was human. We call participants with a quality score of 12 “high-quality participants”. Across our entire sample of 906 participants, we identified 171 LQPs and 182 HQPs. While we identified 171 potential bots (18.9% of the sample), Google’s reCAPTCHA V3 identified a mere 16 potential bots (1.8% of the sample). Specifically, this means that these 16 individuals received a score from reCAPTCHA V3 below 0.5 (the threshold commonly used as a cutoff on many platforms, including Qualtrics). Notably, our bot-score metric agrees with the designation of potential bot for some, but not all, of these 16 individuals. Using our quality score methodology, these 16 consist of: five participants with a quality score of 0, three participants with a quality score of 1, three with a quality score of 5.5, and one participant each at quality scores of 6, 6.5, 9, 10, and 10.5. The three highest scorers of these 16 potential bots all had reCAPTCHA scores of 0.4 which is barely below the 0.5 threshold used to commonly identify bots using reCAPTCHA V3. Thus, we believe it is likely that these individuals are humans who happened to have browsing histories that were deemed more bot-like (e.g., if these individuals had recently cleared their browsing history and cookies, then their relative lack of history could look more bot-like to reCAPTCHA V3). A full distribution of participants by our quality score is shown in Fig. 2 below.

It is worth noting that, while we aimed to identify bots, it could be the case that some of the members of the low-quality participant (LQP) category are simply lazy, inattentive, and/or non-English speakers. For instance, a response that is simply a string of seemingly random numbers or single word answers could easily have been provided by humans that are not taking the survey seriously or do not understand the question. When examining response lengths LQPs and HQPs look very different. LQPs average response length was 23.7 (SD = 43.9) characters, and HQPs average response length was 69.1 (SD = 36.9) characters. This difference was significant (*t*(1340) = 21.1, *p* < 0.05). However, LQPs took an average of 37.8 (SD = 113.6) s to respond, and HQPs took an average of 43 (SD = 41.8) s. This difference is not significant (*t*(848) = 1.13, *p* = 0.26). This means that LQPs were taking about as long as HQPs to write far less. Thus, this is inconsistent with LQPs being entirely made up of lazy individuals trying to get through the survey as possible. It is more likely that LQPs are bots trained to delay their page submission, people who are distracted, or people who are non-native English speakers. For example, the individual who wrote “SNOW” for the Ice-Feet question took 17.8 s to do so. Given the lackadaisical nature of this response, it is quite peculiar that it took them so long to submit their response. Ultimately, for the purposes of ensuring data quality in online social science research, there is value in screening LQPs out of a survey regardless of why they are low quality.

While we focus on LQPs and HQPs throughout this paper, it is also worth considering the performance of intermediate scorers. A score can be intermediate for two reasons: (1) the respondent may have given a valid response to at least one but not all four of the identification questions (e.g., the coders unanimously agreed that a response for one of the identification questions was human, but unanimously agreed that the other responses were non-human) or (2) the respondent may have no valid responses (e.g., the coders were inconsistent and/or unconfident for all four of the identification questions). If intermediate participants fall into the first group, then it is stronger evidence that they may be a lazy human who put forth effort on one but not all four identification questions. Membership in the second group is much more ambiguous. Applying this logic, we believe that many of our intermediate scorers are likely humans. As Table 1 indicates, many of the intermediate scores belong to the first group and are more likely to be human.

**Table 1:** Percentage of participants that received a perfect score on at least 1 of the 4 bot identification questions

Quality score	Percentage received at least 1 perfect score
0	0%
0.5	0%
1	0%
1.5	0%
2	0%
2.5	0%
3	41%
3.5	25%
4	25%
4.5	38%
5	42%
5.5	79%
6	86%
6.5	80%
7	100%
7.5	100%
8	100%
8.5	100%
9	94%
9.5	100%
10	100%
10.5	100%
11	100%
11.5	100%
12	100%

Table  1 shows, for a given quality score, what percentage of participants had a perfect score on at least one of the identification questions. This means that for at least one of the four questions, the raters unanimously agreed the response looked human. For example, the table indicates that of the participants who received a quality score of three, 41% of them received that score from a single question and unequivocally failed the other three questions. Low-quality scores represent more bot-like, lower-quality participants with a quality score = 0 corresponding to an LQP. High-quality scores represent more human-like, higher-quality participants with a quality score = 12 corresponding to an HQP.

### Bot-detection questions

Our goal was to create automated, scalable, valid, and logistically viable upfront bot screening questions. To that end, we developed nine different types of questions that cover six distinct categories of potential attention checks0F^1^: (1) Association (two question types), (2) Learning (one question type), (3) Theory of Mind (two question types), (4) Identify-Sort-Add (one question type), (5) Image Processing & Reasoning (two question types), and (6) Personal references (one question type). In order to cut down on survey length on the participant’s end, we displayed each individual with a subset of the total number of questions in each category. Many of these questions are multiple choice in nature, but some require correct entry of a number or single word. Table 2 summarizes the performance of humans and bots by overall question type. (Our dataset and study materials are accessible at the following OSF link: https://osf.io/x5wzh/?view_only=e31082fa3e224959a5da2101f2ba10ac).

**Table 2:** Low-quality participants and high-quality participants performance by question type

	LQPs	HQPs	Chance
Association_a_	48.8%	99.7%	25%
Association_b_	36.3%	93.2%	25%
Learning	25.1%	83.8%	0%
Theory of Mind_a_	32.5%	93.2%	25%
Theory of Mind_b_	9.4%	83.1%	0%
Identify, Sort, Add	10.8%	87.1%	0%
Image Processing & Reasoning_a_	51.2%	98.4%	25%
Image Processing & Reasoning_b_	54.6%	99.5%	25%
Personal References	38.3%	98.4%	25%

Table  2 compares the percentage of time that LQPs and HQPs answered questions of a given category correctly. These success rates are compared with the chance rate (the likelihood participants would answer correctly by random chance). Questions with a chance rate of 25% correspond to four-answer multiple choice questions. Questions with 0% correspond to free-response questions.

### Association_a_

Methods: Association_a_ questions rely on people’s ability to select an object that displays the most (or least) of a particular descriptor from a set of distractors. For instance, a question may ask participants:

- “Which of the following is generally the loudest?”

• Car horn (1)*
• Chili oil (2)
• Socks (3)
• Buffalo sauce (4)

In order to create scalable versions of this sort of question, we compiled a database of 20 nouns that are commonly associated with a given adjective (e.g., loudness) and sets of words that exhibit that adjective (e.g., car horn). For each question, we randomly sample an adjective, and randomly sample a correct answer from the appropriate set. The rest of the answer choices are randomly sampled from other sets of words. While advanced NLP algorithms such as ChatGPT may be able to solve a question like this, more common “Googler” bots will struggle with a question like this. When generating the sets of words, it was our aim to find nouns that would be clearly associated with the adjective compared to the other answer choices. However, we did not want these associations to be so strong such that bots could easily and trivially answer the question.

In this study, we explored five Association_a_ questions. Each participant was shown a random subset of two of these questions. For each question, answer choices were presented in random order.

Results: HQPs answered Association_a_ questions correctly 99.7% of the time, and LQPs answered these questions correctly only 48.8% of the time. Both performances are above chance (25%). A more thorough performance breakdown is shown in Table 3.

**Table 3:** Association_a_ performance by quality score

Question	Quality Score = 0 (LQP)	Quality Score [0.5, 3]	Quality Score [3.5, 5.5]	Quality Score [6, 8.5]	Quality Score [9, 11.5]	Quality Score = 12 (HQP)
Association_a1_	59.4%	78.5%	94.6%	96.3%	100%	100%
Association_a2_	52.6%	64.3%	84.8%	86.7%	95.4%	100%
Association_a3_	27.0%	42.5%	63.3%	65.5%	90.6%	98.5%
Association_a4_	41.9%	66.3%	94.3%	96.4%	98.5%	100%
Association_a5_	71.4%	63.8%	97.4%	92%	100%	100%
Association_a_ All	48.8%	64.1%	87.9%	86.6%	96.6%	99.7%

The above table shows performance for participants on Association_a_ questions by groups of quality scores. Low-quality scores represent more bot-like, lower-quality participants with a quality score = 0 corresponding to an LQP. High-quality scores represent more human-like, higher-quality participants with a quality score = 12 corresponding to an HQP. Note these questions were multiple choice with a chance success rate of 25%.

It is worth highlighting the LQP performance on Association_a3_ and Association_a5_. Association_a3_ asked participants to select the most “adventurous” option, for which the correct answer from the list was “snorkeling.” While snorkeling is certainly the most adventurous choice out of a set including: “Cheetos, tires, and headaches,” it is more generally likely not to be considered particularly adventurous in isolation. On the other hand, Association_a5_ asked participants to select the “sharpest” option, for which the correct answer was “swords.” Without naming the rest of the set, it is evident that swords will be strongly associated with the adjective sharp. Thus, bots utilizing semantic information and perhaps even “Googlers” were able to get this question correct at higher rates. At the core of these questions is the strength of semantic association for the adjective-noun pairs, where, counterintuitively, *weaker* semantic associations lead to more successful bot-checks. While the present study used random sampling of adjectives and answer choices, future iterations of this approach could use databases of options designed to make it harder on bots that primarily rely on semantic associations.

### Association_b_

Method: Association_b_ questions begin by defining three groups of colors (primary, secondary, and greyscale) in case participants are unfamiliar with these groupings. Next, participants are asked to select a set of objects that has members which are typical of the requested group of colors from a list of possible objects. An example of an Association_b_ question is:

- “Let us define three groups of colors.
- Primary colors: red, yellow, and blue
- Secondary colors: orange, purple, and green
- Greyscale colors: white, grey, and black

Which of the following sets contain objects that are generally the three primary colors^2^?

- Sky, Stop sign, Corn (1)*
- Silver, Flour, Ink (2)
- Bone, Beet, Butter (3)
- Sapphire, Cactus, Cabbage (4)”

Association_b_ questions were created by compiling a database of nouns associated with various colors and randomly sampling from those nouns in order to create answer choices. In this study, there were five Association_b_ questions: Association_b1_ and Association_b2_ ask about primary colors, Association_b3_ and Association_b4_ ask about secondary colors, and Association_b5_ asks about greyscale colors. Each participant was shown a random subset of two of these questions. For each question, answer choices were presented in random order.

Results: Overall, HQPs answered Association_b_ questions correctly 93.2% of the time while LQPs answered Association_b_ questions correctly only 36.3% of the time. Both are well above chance (25%). A full performance breakdown is shown in the Table 4.

**Table 4:** Association_b_ performance by quality score

Question	Quality Score = 0 (LQP)	Quality Score [0.5, 3]	Quality Score [3.5, 5.5]	Quality Score [6, 8.5]	Quality Score [9, 11.5]	Quality Score = 12 (HQP)
Association_b1_	43.7%	58.8%	76.5%	64.3%	85.5%	97.6%
Association_b2_	44.3%	47.4%	73.0%	78.6%	93.4%	98.8%
Association_b3_	50.0%	52.2%	64.7%	69.6%	74.6%	85.2%
Association_b4_	23.6%	30.2%	52.8%	55.9%	78.9%	89.6%
Association_b5_	21.7%	44.9%	66.7%	69.0%	89.3%	93.5%
Association_b_ All	36.3%	46.8%	66.7%	66.9%	84.1%	93.2%

The above table shows performance for participants on Association_b_ questions by groups of quality scores. Low-quality scores represent more bot-like, lower-quality participants with a quality score = 0 corresponding to an LQP. High-quality scores represent more human-like, higher-quality participants with a quality score = 12 corresponding to an HQP. Note these questions were multiple choice with a chance success rate of 25%.

As Association_b4_ and Association_b5_ show LQP performance at about chance (25%), and HQP performance above 89.6% for the two questions, the diagnosticity of these two questions appears to be particularly strong. However, it is unclear what is responsible for this increase in diagnosticity, as Association_b4_ is conceptually the same as Association_b3_, but shows markedly different results (to see text for these and other questions, please see our Online Supplemental Materials).

Future research could explore variations of this question type using arbitrary, fictional color groupings (e.g., “Jabberwocky colors: Blue, Black, Pink”), which may prove particularly difficult for bots to decipher. However, it is an empirical question as to whether or not this additional learning-based component to Association_b_ questions will indeed hinder bot performance without harming human performance.

### Learning

Methods: Learning questions require language skills, pattern recognition, and deductive inference. Participants are told that they have discovered a Rosetta Stone allowing the translation of ancient recipes to English. Each recipe names ingredients, in both “Primeval Grekke” and English, noting how much of each ingredient should be added. However, there is a word missing on both the Primeval Grekke and English side. These blanks require using context to translate a word. Participants were asked both inference questions and a simple reading comprehension question concerning the content of the recipe, for example:

“While excavating an ancient kitchen site, we discovered some phrases in Primeval Grekke. We also found a nearby tablet with the phrases’ English translations but some words are no longer visible.

**Fig. 3:**
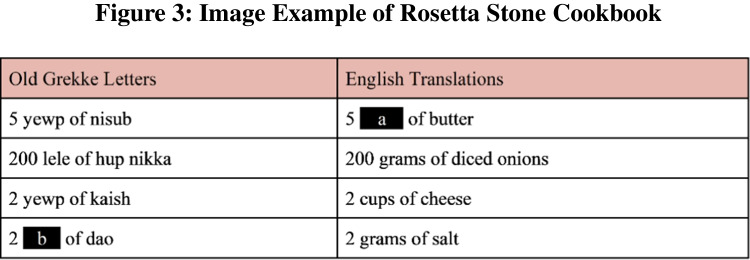
Image example of Rosetta Stone cookbook

Figure 3 shows an example of the recipe translation task that participants faced during Learning questions.

- What is the correct English translation in blank (a)? [Free-response] *cups
- What is the correct ancient Grekke word in blank (b)? [Free-response] *lele
- How many cups of cheese does the recipe call for? [Free-response] *2”

To compile the database of stimuli, a list of 25 English words and 25 non-words was created. For each stimulus created, a random English culinary word was paired with a corresponding Grekke word which was randomly selected from the set of non-words. In total, there were five images of ancient recipes, each with three sub-questions about the image. Participants were shown a random subset of two of these images.

Results: HQPs answered these questions correctly 83.8% of the time, and LQPs answered these questions correctly 25.2% of the time. This was largely driven by particularly high success rates at reading comprehension questions. LQPs successfully translated Grekke into English only 10.2% of the time, and successfully translated English into Grekke 12.3% of the time, but were able to successfully answer reading comprehension questions 52.9% of the time. HQPs successfully translated into English 80.8% of the time, successfully translated into Grekke 72.1% of the time, and successfully answered reading comprehension questions 98.4% of the time. A breakdown by question and question type are shown in Table 5.

**Table 5:** Learning performance by quality score

Question	Quality Score = 0 (LQP)	Quality Score [0.5, 3]	Quality Score [3.5, 5.5]	Quality Score [6, 8.5]	Quality Score [9, 11.5]	Quality Score = 12 (HQP)
Grekke to English	10.2%	22.2%	50.0%	56.3%	57.4%	80.8%
English to Grekke	12.3%	30.0%	54.0%	64.8%	57.1%	72.1%
Reading Comp	52.9%	71.1%	86.8%	92.3%	97.3%	98.4%
Learn All	25.1%	41.1%	63.6%	71.1%	70.6%	83.8%

The above table shows performance for participants on Learning Type questions by groups of quality scores. Low-quality scores represent more bot-like, lower-quality participants with a quality score = 0 corresponding to an LQP. High-quality scores represent more human-like, higher-quality participants with a quality score = 12 corresponding to an HQP. Note the first two parts (part a and b) of these questions were free response and the third part (part c) of these questions were multiple choice with a chance success rate of 25%.

### Theory of Mind_a_

Method: Theory of Mind_a_ questions follow a vignette structure in which giving a correct answer relies on proper application of simple logic and perspective taking. These written vignettes parallel the initial visual paradigms that were used in early work to better understand the development of theory of mind in children (cf., Wimmer & Perner, 1983). In these early studies, children saw a sketch that showed a protagonist placing an object in location *x* (e.g., the kitchen). Subsequently the protagonist leaves. While they are away, another character enters and moves the object from location *x* to location y (e.g., a bedroom). Children are then asked to point where the protagonist is going to look for the object. Children 3–4 years old tend to fail to answer this question correctly, indicating the protagonist will look for the object in location *y*. On the other hand, the vast majority of children 6–9 years old are able to understand that the protagonist has a different, incorrect information set of information about the location of the object and successfully indicate the protagonist will look in location *x*. An archetypal question for this category is shown below:

“Tommy was in the kitchen playing with his toy car while his mom cooked dinner for a party outside. After playing for a little bit, he puts his toy on the floor, and goes into the garage to play. While Tommy is outside, his mom notices the toy on the floor, and she puts it in Tommy’s room. Immediately after playing, where does Tommy think his toy is?

• The kitchen (1) *
• His room (2)
• Outside (3)
• The garage (4)”

These questions seek to lean on adults’ ability to understand the perspective of people with different information sets to themselves. Simultaneously, these questions seek to hinder bot performance with the inclusion of a specific lure, an answer that would be given by someone who fails to understand the different information sets. In the above question, this lure is “his room” because that is the true location of the toy (unbeknownst to Tommy). If bots are prone to the same failure in perspective taking found in small children, then we would expect a disproportionate number of bots to answer with the choice “his room.”

In our study, there were five Theory of Mind_a_ questions. Each participant was shown a random subset of two of these questions. For each question, answer choices were presented in random order.

Results: HQPs answered these questions correctly 93.2% of the time, and LQPs answered these questions correctly only 32.5% of the time. A more thorough performance breakdown is given in the Table 6.

**Table 6:** Theory of Mind_a_ performance by quality score

Question	Quality Score = 0 (LQP)	Quality Score [0.5, 3]	Quality Score [3.5, 5.5]	Quality Score [6, 8.5]	Quality Score [9, 11.5]	Quality Score = 12 (HQP)
Theory of Mind_a1_	30.6%	36.9%	50.0%	63.0%	91.8%	95.8%
Theory of Mind_a2_	21.2%	37.3%	61.8%	62.1%	92.1%	100.0%
Theory of Mind_a3_	20.3%	21.8%	21.2%	40.5%	59.6%	84.6%
Theory of Mind_a4_	21.5%	29.5%	36.8%	40.0%	80.7%	85.9%
Theory of Mind_a5_	62.0%	48.0%	60.0%	54.2%	82.5%	97.4%
Theory of Mind_a_ All	32.5%	35.0%	46.0%	51.4%	82.1%	93.2%

The above table shows performance for participants on Theory of Mind_a_ questions by groups of quality scores. Low-quality scores represent more bot-like, lower-quality participants with a quality score = 0 corresponding to an LQP. High-quality scores represent more human-like, higher-quality participants with a quality score = 12 corresponding to an HQP. Note these questions were multiple choice with a chance success rate of 25%.

This question type was largely successful in differentiating between LQPs and HQPs. It is worth noting, however, that while these questions are simple enough to write, their vignette nature requires effort and creativity on the part of the researcher. Distilling these questions into a formula will likely make them more easily solved by bots, but a creative writer should be able to generate questions analogous to those provided here that do not necessarily have a simple formulaic structure. Notably, LQPs often (but not always) fell for the lure answer presented in questions. Table 7 shows the percentage of LQPs and HQPs that selected the lure answer for each of the five questions. Given that not all LQP mistakes were due to the lure answers, and the fact that LQPs answered the questions correctly at rates close to chance (25%), it would be a worthwhile endeavor to explore how frequently bots get these questions right as the number of answer choices increases.

**Table 7:** Theory of Mind type a percentage of LQPs and HQPs selecting the lure answer

Question	LQPs (Quality Score = 0)	HQPs (Quality Score = 12)
ToMA1	44.4%	2.8%
ToMA2	46.2%	0%
ToMA3	63.5%	7.7%
ToMA4	16.9%	2.6%
ToMA5	20.3%	2.4%
ToMA All	38.0%	2.9%

The above table shows the rates that LQPs (quality score = 0) and HQPs (quality score = 12) selected the lure answer in Theory of Mind type A questions. Chance rate of selecting this lure is 25%.

#### Theory of Mind_b_

**Fig. 4:**
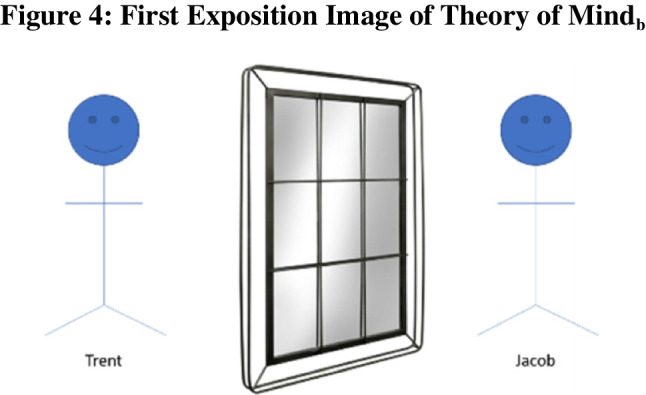
The first image that participants were shown during the exposition of Theory of Mind_b_ questions. This blank window was always the first image shown to participants for this question type

**Fig. 5:**
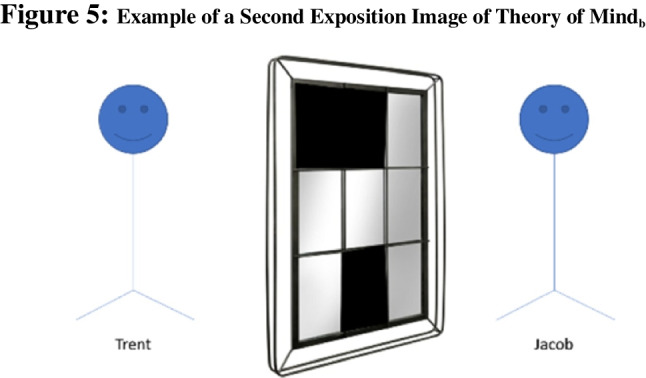
The second image that participants were shown during the exposition of Theory of Mind_b_ questions. The exact panes that are blacked out is randomly determined, and thus, is different question to question

**Fig. 6:**
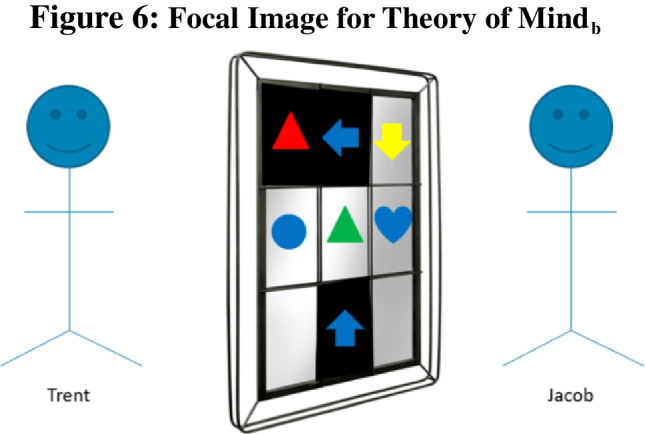
The third image that participants were shown during the exposition of Theory of Mind_b_ questions. It is randomly determined which windowpanes will contain an object. Subsequently the exact shape and color of the object is randomly determined. Participants are then asked question regarding these objects. **a**) How many blue shapes does Trent see? [Free-response] *2. **b**)s How many triangles does Trent see? [Free-response] *1. **c**) What shape does Trent think the arrow is pointed towards? Please select the correct color AND shape. [multiple-choice where participants select from a set of six different colors and 16 different shapes] *Blue heart”

Methods: Similar to Theory of Mind_a_ questions, Theory of Mind_b_ questions rely on adult humans’ perspective taking ability. However, unlike their counterparts, Theory of Mind_b_ questions are image-based, rather than vignette-based. These questions feature two individuals looking at one another through a window, and it asks participants to indicate what can be seen from the viewpoint of one of these lookers, for example :

“Jacob and Trent are currently looking at each other through a window Figs 4, 5, 6.

Jacob and Trent are currently looking at each other through a window. However, now some of the windowpanes are blacked out. When a windowpane is blacked out, neither Trent nor Jacob can see through to the other side through that particular windowpane.

Jacob and Trent are currently looking at each other through a window. However, now some of the windowpanes are blacked out. When a windowpane is blacked out, neither Trent nor Jacob can see through to the other side through that particular windowpane. After the blackout, shapes are added to Jacob’s side of the window as indicated below.

Theory of Mind_b_ questions lend themselves especially well to scaling because different elements of each window can be generated randomly with ease. We randomly blacked out between 2–4 panes such that the majority of panes would always be clear. Either seven or eight items were randomly created from a basic set of six colors and 16 shapes and were randomly placed into each windowpane.

As the given example demonstrates, multiple sub-questions can be asked about the same image. In our study, five distinct images were used. Participants were shown a random subset of two of these images. They answered all three sub-questions for each image, two free response questions (a–b) and one “challenge” multiple choice (c). Challenge sub-questions took one of three forms: (i) requiring participants to identify the referent of an arrow (as in the example above) or (ii) requiring participants to identify the direction an arrow is facing (e.g., the arrow is pointed “up”) or (iii) asking for the number of points a star has when there are multiple stars on screen.

Results: Theory of Mind_b_ questions were difficult for both LQPs and HQPs, especially so on the challenge sub-questions. HQPs answered Theory of Mind_b_ questions correctly 83.1% of the time, and LQPs answered these questions correctly only 9.4% of the time. Both the HQP and LQP performances are greatly deflated by the challenge sub-questions. HQPs answered such challenge questions correctly 77.6% of the time. However, LQPs struggled even more so with these questions, answering correctly only 5.8% of the time. Notably, one of the challenge questions was significantly easier than the rest. This was the question that asked for the number of points of a particular star. Unfortunately, by random chance, the answer to this question was five points, the most common number of points for a star. As a result of this oversight, LQPs answered this particular challenge sub-question correctly 25.8% of the time, and HQPs answered this question correctly 94.9% of the time. Omitting this question, LQPs answered the challenge sub-questions correctly a dismal 1.4% of the time. On the other hand, HQPs answered these questions correctly 68.1% of the time. More detailed results for Theory of Mind_b_ questions are shown in Table 8.

**Table 8:** Theory of Mind_b_ performance by quality score

Question	Quality Score = 0 (LQP)	Quality Score [0.5, 3]	Quality Score [3.5, 5.5]	Quality Score [6, 8.5]	Quality Score [9, 11.5]	Quality Score = 12 (HQP)
Theory of Mind_b_ Free response (a-b)	11.1%	20.4%	39.9%	51.4%	76.9%	85.8%
Theory of Mind_b_ Challenge	5.8%	8.7%	16.2%	31%	57.4%	77.6%
Theory of Mind_b_ All	9.4%	16.5%	32.1%	44.6%	70.4%	83.1%

The above table shows performance for participants on Theory of Mind_b_ questions by groups of quality scores. Low-quality scores represent more bot-like, lower-quality participants with a quality score = 0 corresponding to an LQP. High-quality scores represent more human-like, higher quality participants with a quality score = 12 corresponding to an HQP. Note the first two parts (a–b) of these questions were free response, and the challenge questions were generally required correct selection of both color and shape from a list.

Theory of Mind_b_ questions were incredibly effective bot eliminators. The combination of image processing, application of perspective taking logic, and the free-response nature of many of these questions proved very difficult for LQPs. However, HQPs also made more mistakes in this category than they did in others, especially so in the challenge questions. One potential solution to prevent more humans from being screened out could be to present participants with many sub-questions about the same image (as we did with groups of three). If the screening criteria required getting only two (one) out of three sub-questions correct, then 88.4% (95.0%) of HQPs in our sample would have advanced, while 4.7% (19.9%) of LQPs in our sample would have advanced.

### Identify-Sort-Add

Methods: For Identify-Sort-Add questions, participants are shown a vignette in which many common objects are listed (e.g., groceries). After reading the list, participants are asked how many items on the list belong to a given category. The key to answering these questions correctly is knowledge about individual object category membership. While the types of objects and categories that can be used are widely variable, we unite these questions under a single archetypal format that lends itself to multiple sub-questions based upon a single vignette. An example of such a vignette is shown below.

- “Jane and Ricky both went grocery shopping today, and they are now putting drinks away into the fridge. Jane put 2 bottles of Coke, 1 bottle of milk, 3 bottles of water, and 4 bottles of beer in the fridge. Ricky put 1 bottle of Pepsi, 4 bottles of Dr. Pepper, 2 bottles of water, 3 bottles of wine, and 2 bottles of beer in the fridge.
- How many bottles of alcohol did they put in the fridge? (FR)
- How many bottles of soda did they put in the fridge? (FR)”

Results: This question type proved especially difficult for potential bots to complete correctly, with only 10.8% of LQPs reporting the correct answer. HQPs had a much higher success rate of 87.1% on Identify-Sort-Add questions. A breakdown by quality score is shown in Table 9.

**Table 9:** Identify-Sort-Add performance by quality score

Question	Quality Score = 0 (LQP)	Quality Score [0.5, 3]	Quality Score [3.5, 5.5]	Quality Score [6, 8.5]	Quality Score [9, 11.5]	Quality Score = 12 (HQP)
Identify-Sort-Add all	10.8%	16.5%	38.3%	37.5%	73.4%	87.1%

The above table shows performance for participants on Identify-Sort-Add (ISA) questions by groups of quality scores. Low-quality scores represent more bot-like, lower-quality participants with a quality score = 0 corresponding to an LQP. High-quality scores represent more human-like, higher-quality participants with a quality score = 12 corresponding to an HQP. Note these questions were generally free response with the exception of a single four-answer multiple-choice subpart in question ISA2.

Identify-Sort-Add questions proved slightly more difficult for HQPs than some easier question types but hold immense potential as bot screeners given the low LQP performance. Key to HQP success in this question category is selection of objects and categories that humans are familiar with. For instance, HQPs answered the aforementioned alcohol and soda questions correctly 97% and 96% of the time, respectively. On the other hand, a question that asked about distinct species of animals (a category people may be less familiar with) in a list yielded an HQP success rate of only 84%. On the other hand, LQPs answered these questions correctly 20, 5, and 18% of the time, respectively. Thus, utilizing more commonly identified items does not compromise the question’s ability to screen out bots while giving a significant boost to human performance.

### Image Processing & Reasoning_a_

**Fig. 7:**
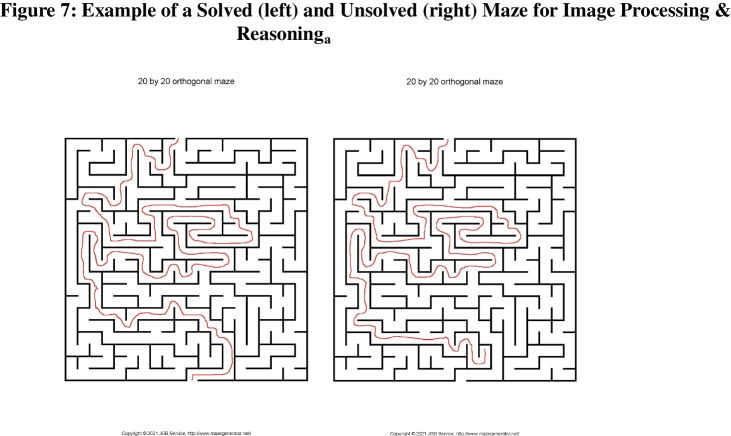
Examples of a solved maze (*left*) and unsolved maze (*right*) that was shown to participants for Image Processing & Reasoning_a_ questions. These mazes were generated on “mazegenerator.net”. The mazes were filled out by hand on a touch screen laptop

Methods: Image Processing & Reasoning_a_ (IPR_a_) questions focus on images of mazes. Specifically, they prompt participants to please select the only (un)successfully completed maze out of the set of mazes presented. In order to create our stimuli, we utilized “mazegenerator.net”. Participants were presented with a set of four mazes, each of which showed a path “attempting to solve” the maze drawn onto it. These maze sets were (1) squares maze in which one must navigate from one side of the square to the other, with one of the four mazes successfully solved (2) circular mazes in which one must navigate from the edge of the circle into the center, with one of the four mazes successfully solved (3) triangular mazes in which one must navigate from the edge of the triangle into the center with one of the four mazes successfully solved and (4) square mazes in which one must navigate from one side of the square to the other, with three of the four maze successfully solved. For question types 1–3, participants were asked to identify which of the four mazes in the set had been successfully solved, and for question type 4 participants were asked to identify which of the four mazes had not been successfully solved. Participants were shown a random subset of two of these question types, and the answer order was randomized for each question. An example maze is shown below Fig. 7.

Results: As expected, HQPs were either familiar with, or were able to intuit completion criteria for each of the mazes. In total, 98.4% of HQPs answered these questions correctly. On the other hand, LQPs answered these questions correctly 51.2% of the time, which is also well above chance (25%), but still considerably worse than HQPs. A thorough performance breakdown is shown in Table 10.

**Table 10:** Image Processing & Reasoning_a_ (IPR_a_) performance by quality score

Question	Quality Score = 0 (LQP)	Quality Score [0.5, 3]	Quality Score [3.5, 5.5]	Quality Score [6, 8.5]	Quality Score [9, 11.5]	Quality Score = 12 (HQP)
IPR_a1_	62.2%	71.3%	93.6%	94.1%	97.2%	100.0%
IPR_a2_	64.9%	67.5%	95.3%	100.0%	98.6%	100.0%
IPR_a3_	47.6%	67.7%	78.0%	82.4%	91.7%	95.7%
IPR_a4_	29.9%	53.7%	76.7%	85.3%	97.1%	98.2%
IPR_a_ All	51.2%	65.4%	86.2%	90.8%	95.9%	98.4%

The above table shows performance for participants on Image Processing & Reasoning_a_ questions by groups of quality scores. Low-quality scores represent more bot-like, lower-quality participants with a quality score = 0 corresponding to an LQP. High-quality scores represent more human-like, higher-quality participants with a quality score = 12 corresponding to an HQP. Note these questions were multiple choice with a chance success rate of 25%.

Both LQPs and HQPs did especially well when selecting a completed maze out of a set of square mazes or circular mazes (IPR_a1_ and IPR_a2_ respectively). The relatively less common triangular maze question (IPR_a3_) was modestly more challenging for HQPs and LQPs. However, the most notable question is IPR_a4_, the question in which participants were asked to select the only incomplete square maze. In this case, LQPs answered correctly 29.9% of the time, only slightly above chance. On the other hand, 98.4% of HQPs answered this question correctly. This suggests that IPR_a4_ is particularly useful as a bot-check, almost perfectly discriminating HQPs from LQPs. That being said, we only had one question that asked participants to identify the incomplete maze, and thus, further stimulus sampling (Monin & Oppenheimer, 2014) would be beneficial to build confidence in this sub-question type’s viability.

A possible way of improving this question type could be to add “cheating” mazes in which the attempted maze solution blatantly crossing the lines. In our initial stimuli, mazes were either completed correctly or they were shown an incomplete solution that finds a conclusion at one of the maze’s dead ends. However, presuming humans could clearly identify the violation of the maze’s rules, a “cheating” maze could make for a potentially attractive lure for identifying the completed maze, and it could make for a potentially shrouded incomplete maze. Lastly, it could be worthwhile exploring the utilization of multiple maze types as answer choices for the same question.

#### Image Processing & Reasoning_b_

**Fig. 8:**
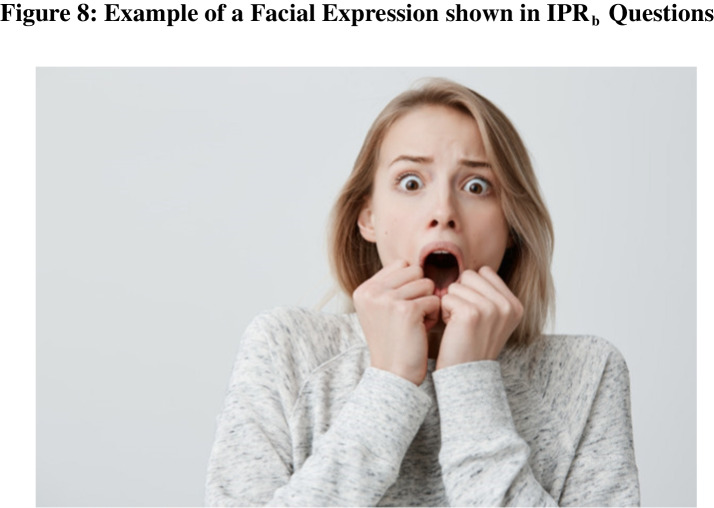
Shows an example of a facial expression shown to participants for IPR_b_ questions. Images for our study were acquired from a variety of stock photo face repositories (e.g., freepik.com)

Methods: Image Processing & Reasoning_b_ (IPR_b_) questions revolve around recognition of human emotions based upon their faces. In general, people have a good ability to recognize emotions based solely upon facial expressions (Carroll & Russell, 1996) and have a good sense of what sorts of events would elicit different emotions (Ekman, 1992). Image Processing & Reasoning_b_ questions took one of two formats. In the first format, participants are shown an image of a facial expression and asked to select the event that was most likely to have occurred before the image was taken based upon the expression on their face. Thus, in order to answer this question successfully, participants must correctly synthesize what emotion the face is showcasing and what common emotional reactions would be for each event listed. The second format of these questions simply inverted the event and faces. Participants are told an event that occurred, and then they are asked to select the face of the individual that is congruent with that event having happened. Images for our study were acquired from a variety of stock photo face repositories (e.g., freepik.com). For each basic emotion, we generated 20 events that could lead to said emotions. These events were sampled randomly. An example question is shown below Fig. 8.

• She baked a loaf of bread. (1).
• She watched someone put their hand into a bucket of spiders. (2)*
• She got a promotion at work. (3).
• She had a good night sleep. (4)”.

In our study, each participant was randomly shown one of each type of Image Processing & Reasoning_b_ question. For each question, answer choices were presented in random order.

Results: HQPs answered Image Processing and Reasoning_b_ questions correctly 99.5% of the time. LQPs answered these questions correctly 54.7% of the time, which is well above chance (25%). A full breakdown by question is shown in Table 11.

**Table 11:** Image Processing & Reasoning_b_ (IPR_b_) by quality score

Question	Quality Score = 0 (LQP)	Quality Score [0.5, 3]	Quality Score [3.5, 5.5]	Quality Score [6, 8.5]	Quality Score [9, 11.5]	Quality Score = 12 (HQP)
IPR_b1_	57.3%	68.4%	89.7%	90.1%	99.3%	100.0%
IPR_b2_	52.0%	59.1%	89.7%	87.3%	95.3%	98.9%
IPR_b_ All	54.7%	63.7%	89.7%	88.7%	97.3%	99.5%

The above table shows performance for participants on Image Processing & Reasoning_b_ questions by groups of quality scores. Low-quality scores represent more bot-like, lower-quality participants with a quality score = 0 corresponding to an LQP. High-quality scores represent more human-like, higher-quality participants with a quality score = 12 corresponding to an HQP. Note these questions were multiple choice with a chance success rate of 25%.

This suggests that potential bots have a relatively higher capacity to read emotional states from faces than we had initially expected. This may be the case because the images we curated came from public face repositories on the internet (e.g., freepik.com). This allowed us to use validated images that are known to showcase a discernable emotion. However, constructing our collection of faces in this way leaves the question type vulnerable to reverse-image searches. Further, an oversight on our part led to file names of the photos containing the name of the emotion being displayed. Specifically, upon inspecting the properties of the image, one would be able to find the images initial file name which often included the emotion being displayed. Thus, bots may have been reading the file names allowing them to identify the appropriate emotion. Future work could create a novel repository of images for which there is no trail on the Internet and with arbitrary or incorrectly labeled file names which may increase this question type’s ability to discriminate between bots and humans.

Another possible way to increase the efficacy of these questions would be to display 4 images and 4 text descriptions to participants. Then participants would be asked to match each image with the correct description. This should add minimal difficulty for humans, but it is likely to hinder bot performance. Bots would be far less likely to answer the question correctly by chance. This would also ensure that the question could only be solved by comparing the pictures and descriptions, and not solely processing the descriptions alone (e.g., selecting the longest description or the description with the most unusual words). Future work could explore how much of an extra hindrance this question structure would be to bots.

#### Personal Reference

Method: Personal Reference questions utilize a participant’s prior answers to shroud the meaning of simple questions. Participants are asked about a preference or personal history, and then are asked a follow-up question that requires the answer to the previous question to be answered accurately. For instance:

## Opinion question:

“Which of the following locations would you most want to visit?

• Washington DC (1)
• Giza, Egypt (2)
• Paris, France (3)
• Iceland (4)”

## Focal diagnostic question:

“Which of the following is most closely associated with the location you most want to visit (from the previous question)?

• The White House (1)
• Pyramids (2)
• The Eiffel Tower (3)
• Ice caves (4)”

A question explicitly asking, “which of the following is most closely associated with Paris?” is likely to be trivial for bots, either through Internet search or more complex semantic associations using NLP. However, the two-part nature of Personal Reference questions shrouds the focal question, making it more difficult for bots that use semantic associations or Google, while presumably not affecting a human participant’s ability to answer the focal question.

The focal question does not provide bots any content to semantically associate or Google without referring to storage of prior answers. While the first, opinion-based question has no correct answer, the second, focal question does have a correct answer, dependent on the selection in the first question. These questions are trivially scalable because the first question can be stylized in many ways with ease, such as “Which of the following is your favorite _____?” Moreover, these questions do not require participants to have prior knowledge of each answer choice (e.g., if someone were to pick their favorite snack out of a list, they would presumably not pick a snack that they are unfamiliar with).

In this study, there were four Personal Reference questions pairs. Each participant was shown a random subset of two of these questions pairs. For each, participants were asked the opinion question first, then on the next page, participants were asked the second, focal question. Answer choices were presented in random order for each of the questions.

Results: HQPs answered these questions correctly 98.4% of the time. On the other hand, LQPs only answered these questions correctly 38.3% of the time. A more thorough performance breakdown is given in Table 12.

**Table 12:** Personal Reference Performance by quality score

Question	Quality Score = 0 (LQP)	Quality Score [0.5, 3]	Quality Score [3.5, 5.5]	Quality Score [6, 8.5]	Quality Score [9, 11.5]	Quality Score = 12 (HQP)
Personal Reference_1_	48.4%	57.9%	73.9%	83.3%	92.5%	98.9%
Personal Reference_2_	32.1%	39.6%	35.1%	64.5%	89.7%	96.7%
Personal Reference_3_	42.1%	56.7%	78.0%	91.9%	98.4%	100.0%
Personal Reference_4_	30.9%	47.8%	80.0%	81.6%	87.9%	98.0%
Personal Reference All	38.3%	50.8%	68.4%	81.0%	91.9%	98.4%

The above table shows performance for participants on Personal References questions by groups of quality scores. Low-quality scores represent more bot-like, lower-quality participants with a quality score = 0 corresponding to an LQP. High-quality scores represent more human-like, higher-quality participants with a quality score = 12 corresponding to an HQP. Note these questions were multiple choice with a chance success rate of 25%.

It is worth noting that the opinion question and focal question do not necessarily have to be on consecutive pages, although in this study they were. Presumably, the greater the distance between the opinion question and the focal question the harder the question would become.

## General discussion

Our work has confirmed, in an important, naturalistic context, that bots are capable of fooling Google’s reCAPTCHA V3, one of the most encountered CAPTCHAs on the web. While the CAPTCHA identified 1.7% of our sample to be likely bots, our identification questions identified 18.9% of our sample to be LQPs. While it is possible that some of our bot-like participants were actually humans who did not speak English or who put in so little effort that their answers were consistently rated non-human by multiple expert coders, it is probable that many of our LQPs were indeed bots. Our automated bot checks posed a greater challenge to presumed-bots than reCAPTCHA V3; at worst about half of the presumed-bots were detected and at best presumed-bots performed at chance. Given this, we strongly encourage researchers to engage in better data validation and participant screening techniques to ensure data sanctity.

At the time of this paper’s initial writing, one of the best ways to detect bots is using hand-coded, image-based, causal-reasoning, free-response questions. While manually coding these is effortful, it builds confidence that you are dealing with humans. However, such an approach is slow, inefficient, expensive, and can only detect bots post hoc. As such, the use of automated bot checkers is more practical in many situations. Of course, careful consideration should be given when selecting the type of question to be used. Different contexts may lend different utility function for the tradeoff between type I (identifying a human as a bot) and type II (identifying a bot as a human) errors.

We know that the war with bots is ever evolving. We hope that our bot checks can be used as ammunition in this war, but we know that at some point they will become obsolete. However, it is our hope that even as our bot checks become less useful with time, they will inspire researchers to develop more of their own bot checks, whether by utilizing variations of ours or novel questions that are generated in response to a greater awareness and understanding of bots’ presence and algorithms.

Indeed, *during the writing of this paper,* OpenAI released the chatbot ChatGPT which represents a major advance in NLP capabilities. Subsequently, *during the revisions of this paper,* GPT-4 was released, and while ChatGPT was initially not capable of image processing, now it can even process images. Due to the prior inability to submit queries with images, we posed each of our automated checks to GPT-3.5 in January 2023 to get a glance at how they fair against the next step of chatbots’ evolution. However, even more recently (October 2023), we returned to test GPT-4’s capabilities. Table 13 shows ChatGPT’s performance on each question category for which it could provide an answer. Each question was asked to each ChatGPT version one time.

**Table 13:** LQP & HQP performance by question type

	LQPs	HQPs	Chance	GPT-3.5	GPT-4
Association_a_	48.8%	99.7%	25%	100%	100%
Association_b_	36.3%	93.2%	25%	0%	100%
Learning	25.2%	83.8%	0%	NA	80%
Theory of Mind_a_	32.5%	93.1%	25%	80%	80%
Theory of Mind_b_	9.4%	83.1%	0%	NA	13.3%
Identify, Sort, Add	10.8%	87.1%	0%	10%	100%
Image Processing & Reasoning_a_	51.2%	98.4%	25%	NA	25%
Image Processing & Reasoning_b_	54.6%	99.5%	25%	NA	100%
Personal References	38.3%	98.4%	25%	NA	100%

Table  13 compares the percentage of time that LQPs and HQPs answered questions of a given category correctly. These success rates are compared with the chance rate (the likelihood participants would answer correctly by random chance). In addition, this table includes the rates with which ChatGPT answered the questions correctly. Questions with a chance rate of 25% correspond to four-answer multiple choice questions. Questions with 0% correspond to free-response questions.

As shown in the table, GPT-3.5’s performance had already rendered Association_a_ questions obsolete. Association_b_ posed a difficult challenge for GPT-3.5, but GPT-4 easily overcame the difficulties that its predecessor had with this question type. As NLP models advance further, simple association questions may not be adequate bot checks. However, such questions were able to fool what was once a cutting edge chatbot. Thus, these questions are likely still viable options to stump less sophisticated bots. Theory of Mind_a_ questions proved relatively easy for both GPT-3.5 and GPT-4. This finding contributes to the growing literature exploring the performance of large language models (LLMs) on theory of mind tasks (cf., Shapira et al., 2023a). These authors survey this literature as well as perform their own replication studies, and they find that while some LLMs perform well on some theory of mind tasks, this is not robust across all LLMs and all theory of mind tasks. For instance, the flan-tx-xx1 model completed the TriangleCOPA (Gordon, 2016) theory of mind task with 96% accuracy. On the other hand, GPT-4 completed the Faux-Pas-EAI (Shapira et al., 2023b) task with only 27% accuracy.

Indeed, Theory of Mind_b_ questions prove that ChatGPT is not yet equipped to handle all perspective taking tasks, particularly those involving visual cues. For Identify-Sort-Add questions, GPT-3.5 made a variety of mistakes, sometimes mis-categorizing items, and bizarrely, sometimes incorrectly summing the correctly categorized items. GPT-4 ironed out these kinks for these question types. Image Processing & Reasoning_a_ questions proved difficult for the GPT-4 because the chatbot seemed confused as to what the exact success conditions for a maze are. However, should GPT-4 learn these conditions, we have little doubt that it would eventually be able to solve these questions with relative ease. Image Processing & Reasoning_b_ questions were trivial for GPT-4, indicating that the chatbot is very capable of translating facial expressions to emotions and deducing the logic of what scenario may lead to a particular emotion. GPT-3.5 was unable to answer these questions because the chatbot kept getting hung up on the fact that “as a chatbot, it does not have favorite objects”. While GPT-4 gave a similar response at first, we were able to have it “pretend” it had a favorite, after which it was able to easily answer these questions (which is unsurprising given that GPT-4 aced the Association category of questions).

GPT-4 performed impressively across all our question categories with exception of Theory of Mind_b_ and Image Processing & Reasoning_a_. However, most shocking was GPT-4’s performance on our identification questions. In response to the identification question concerning what would happen to the ice-feet on a warm, sunny day, GPT-4 stated: “On a warm, sunny day, the snow sculpture resembling a foot is likely to melt and diminish in size, turning into water that will puddle on the ground.” While a human may not detail each step so specifically, this response would have certainly been coded as human-like in our framework. The responses to the other three questions were similarly human-like. Thus, GPT-4 has shown that the once most surefire bot identification method of asking free-response questions involving causal reasoning and image processing is now far from surefire.

The rapid progress made by GPT-4 is shocking to say the least. However, the ability to give human-like answers to these questions is consistent with other current research that has shown that ChatGPT exhibits many of same biases as humans, including suboptimal strategies in social games (e.g., cooperating in prisoner’s dilemmas) and cognitive biases (e.g., exhibiting the use of the availability heuristic by guessing there are more English words with ‘l’ as the first letter than there are English words with ‘l’ as the third letter) (Azaria, 2023). The level of “humanness” apparent in GPT-4 is incredible. However, we believe this does not devalue the current work. To the contrary, now that the previous gold standard of manual human coding of short answer causal and predictive questions can no longer effectively detect bots, the two successful methods identified in this manuscript (Theory of Mind b and Maze processing) are now (to our knowledge) among the very best tools that have been identified for detecting bots. Moreover, we hope that this work draws attention to AI’s ever-increasing ability to appear human to inspire generations of future researchers to increase the efficacy of bot checks.

### Increasing efficacy

We believe that for our multiple-choice questions, bot performance could be hindered by increasing the number of answer choices, without also hurting human performance. Bot performance being hindered naturally follows from the fact that any bots answering randomly will get the questions correctly by chance less frequently. Given that humans got these questions correct at very high rates, these rates are unlikely to drop with the addition of more answer choices. For instance, increasing the number of mazes presented will not make a participant forget what a completed maze looks like.

### Low-quality participant identities

While we acknowledge that it could be the case that we sometimes identify non-English speaking, and/or inattentive participants as LQPs, we can say with more certainty that many of these participants are not who/what they say they are. This can be seen most strikingly by considering the demographic differences between LQPs and HQPs, specifically the reported education level. In our sample, LQPs reported that they were much more highly educated than HQPs with 93.6% of bots report receiving at least a bachelor’s while only 55.4% of HQPs indicate receiving a bachelor’s. However, across all questions in our survey, LQPs answered our bot-screeners correctly 25.7% of the time and HQPs answered the same questions correctly 89.3% of the time. We see no reason why the better educated group would perform worse on such simple questions. That said, it would be a mistake to use self-reported demographics as a bot-screener, both because it is such a noisy measure, and because, as with spoofing browser history to appear more human-like (Sivakorn et al., 2016), self-reported demographics are easy to spoof.

## Conclusion

While advances in AI have changed our world largely for the better, there will always exist bad actors who wish to use bots for malicious purposes. It is imperative that we continue to improve upon methods to continue to keep bots in check. Whether one’s goal is to improve the quality of research in the social sciences, prevent the spread of misinformation online through social networking platforms, or even making sure there are enough Taylor Swift tickets for her fans, improving bot detection methods can reduce the prevalence of damaging online behaviors.

## Data Availability

Our dataset and study materials are accessible at the following OSF link: https://osf.io/x5wzh/?view_only=e31082fa3e224959a5da2101f2ba10ac.

## Footnotes

1 A seventh category, psychometric, was our least successful and is unreported for brevity’s sake. Materials regarding this category can be found at the following link: https://osf.io/x5wzh/?view_only=e31082fa3e224959a5da2101f2ba10ac

2 A reviewer pointed out that this question wording could be confusing to subjects. While HQPs tended to do well on this question, a slight re-wording to potentially make this question clearer could ask participants: “Which answer choice highlights objects that are generally associated with primary colors?”.

## References

[CR1] Azaria, A. (2023). ChatGPT: More Human-Like Than Computer-Like, but Not Necessarily in a Good Way. *The 45th Annual Meeting of the Cognitive Science Society.*

[CR2] Bessi, A., & Ferrara, E. (2016). Social bots distort the 2016 US Presidential election online discussion. *First Monday*, *21*(11–7).

[CR3] Carroll, J. M., & Russell, J. A. (1996). Do facial expressions signal specific emotions? Judging emotion from the face in context. *Journal of Personality and Social Psychology,**70*(2), 205. 8636880 10.1037/0022-3514.70.2.205

[CR4] Chmielewski, M., & Kucker, S. C. (2020). An MTurk crisis? Shifts in data quality and the impact on study results. *Social Psychological and Personality Science,**11*(4), 464–473. 10.1177/1948550619875149

[CR5] Davis, C. A., Varol, O., Ferrara, E., Flammini, A., & Menczer, F. (2016, April). Botornot: A system to evaluate social bots. In *Proceedings of the 25th international conference companion on world wide web* (pp. 273–274).

[CR6] Ekman, P. (1992). Facial expressions of emotion: an old controversy and new findings. *Philosophical Transactions of the Royal Society of London. Series B: Biological Sciences*, *335*(1273), 63–69. 10.1098/rstb.1992.0008 1348139

[CR7] Ferrara, E., Varol, O., Davis, C., Menczer, F., & Flammini, A. (2016). The rise of social bots. *Communications of the ACM,**59*(7), 96–104. 10.1145/2818717

[CR8] Godinho, A., Schell, C., & Cunningham, J. A. (2020). Out damn bot, out: Recruiting real people into substance use studies on the Internet. *Substance Abuse,**41*(1), 3–5. 31821108 10.1080/08897077.2019.1691131

[CR9] Goodfellow, I. J., Bulatov, Y., Ibarz, J., Arnoud, S., & Shet, V. (2013). Multi-digit number recognition from street view imagery using deep convolutional neural networks. *arXiv preprint. *Retrieved August 1, 2023 from arXiv:1312.6082

[CR10] Gordon, A. (2016). Commonsense interpretation of triangle behavior. In *Proceedings of the AAAI Conference on Artificial Intelligence*, *30*(1)

[CR11] Guerar, M., Verderame, L., Migliardi, M., Palmieri, F., & Merlo, A. (2021). Gotta CAPTCHA’Em all: A survey of 20 years of the human-or-computer dilemma. *ACM Computing Surveys (CSUR),**54*(9), 1–33. 10.1145/3477142

[CR12] Huang, S. Y., Lee, Y. K., Bell, G., & Ou, Z. H. (2008, July). A projection-based segmentation algorithm for breaking MSN and YAHOO CAPTCHAs. In *Proceedings of the World Congress on Engineering* (Vol. 1).

[CR13] Litman, L., Robinson, J., & Abberbock, T. (2017). TurkPrime.com: A versatile crowdsourcing data acquisition platform for the behavioral sciences. *Behavior Research Methods*, *49*(2), 433–442. 10.3758/s13428-016-0727-z PMC5405057 27071389

[CR14] Lynas, G.A., *Two Feet of Snow*. [sculpture]. New York, NY.

[CR15] Monin, & Oppenheimer, D. M. (2014). The limits of direct replications and the virtues of stimulus sampling *Social Psychology*, *45*(4), 299–300.

[CR16] Moss, A.J., & Litman, L. (2018). After the bot scare: Understanding what’s been happening with data collection on MTurk and how to stop it. *CloudResearch Blog*. Retrieved August 1, 2023 from https://www.cloudresearch.com/resources/blog/after-the-bot-scare-understanding-whats-been-happening-with-data-collection-on-mturk-and-how-to-stop-it/

[CR17] Mori, G., & Malik, J. (2003, June). Recognizing objects in adversarial clutter: Breaking a visual CAPTCHA. In *2003 IEEE Computer Society Conference on Computer Vision and Pattern Recognition, 2003. Proceedings.* (Vol. 1, pp. I–I).

[CR18] Sivakorn, S., Polakis, J., & Keromytis, A. D. (2016). I’m not a human: Breaking the Google reCAPTCHA. *Black Hat*, *14*.

[CR19] Shapira, N., Levy, M., Alavi, S. H., Zhou, X., Choi, Y., Goldberg, Y., Sap, M., & Shwartz, V. (2023a). Clever hans or neural theory of mind? Stress testing social reasoning in large language models. Retrieved January 24, 2024 from arXiv:2305.14763.

[CR20] Shapira, N., Zwirn, G., & Goldberg, Y. (2023b). How Well Do Large Language Models Perform on Faux Pas Tests? *In Findings of the Association for Computational Linguistics: ACL,**2023*, 10438–10451.

[CR21] Storozuk, A., Ashley, M., Delage, V., & Maloney, E. A. (2020). Got bots? Practical recommendations to protect online survey data from bot attacks. *The Quantitative Methods for Psychology,**16*(5), 472–481. 10.20982/tqmp.16.5.p472

[CR22] Teitcher, J. E., Bockting, W. O., Bauermeister, J. A., Hoefer, C. J., Miner, M. H., & Klitzman, R. L. (2015). Detecting, preventing, and responding to “fraudsters” in internet research: Ethics and tradeoffs. *Journal of Law, Medicine & Ethics,**43*(1), 116–133. 10.1111/jlme.12200 PMC4669957 25846043

[CR23] Von Ahn, L., Blum, M., Hopper, N. J., & Langford, J. (2003). CAPTCHA: Using hard AI problems for security. In *International conference on the theory and applications of cryptographic techniques* (pp. 294–311). Springer, Berlin, Heidelberg.

[CR24] Webb, M. A., & Tangney, J. P. (2022). Too good to be true: Bots and bad data from Mechanical Turk. *Perspectives on Psychological Science*, 17456916221120027. 10.1177/17456916221120027 36343213

[CR25] Wimmer, H., & Perner, J. (1983). Beliefs about beliefs: Representation and constraining function of wrong beliefs in young children’s understanding of deception. *Cognition,**13*(1), 103–128. 6681741 10.1016/0010-0277(83)90004-5

[CR26] Zhao, B., Weng, H., Ji, S., Chen, J., Wang, T., He, Q., & Beyah, R. (2018, January). Towards evaluating the security of real-world deployed image CAPTCHAs. In *Proceedings of the 11th ACM Workshop on Artificial Intelligence and Security* (pp. 85–96).

